# Evaluation of Age and Sex Differences in Contemporary versus High-Sensitivity Troponin I Measurement in Hospitalized Patients

**DOI:** 10.3390/jcm13082428

**Published:** 2024-04-21

**Authors:** Hussam Alkhalaileh, Ruhan Wei, Ashly Cordero Rivera, Mustafa Goksel, Jason K. Y. Lee, Ernest Mazzaferri, Jr., JoAnna Jones, Jieli Li

**Affiliations:** 1 The Ohio State College of Medicine, The Ohio State University, Columbus, OH 43210, USA; hussam.alkhalaileh@osumc.edu; 2 Department of Pathology, Duke University School of Medicine, Durham, NC 27710, USA; ruhan.wei@duke.edu; 3 Department of Pathology, Wexner Medical Center, The Ohio State University, 410 W 10th Ave., Columbus, OH 43210, USA; ashly.cordero@gmail.com (A.C.R.); mgoksel88@gmail.com (M.G.); joanna.jones@osumc.edu (J.J.); 4 Department of Clinical Laboratory, University Hospital, The Ohio State University, Columbus, OH 43210, USA; jason.lee@osumc.edu; 5 Division of Cardiovascular Medicine, Department of Internal Medicine, Wexner Medical Center, The Ohio State University, Columbus, OH 43210, USA; ernest.mazzaferri@osumc.edu

**Keywords:** troponin, high sensitivity, contemporary, age, sex, hospitalized patients

## Abstract

**Background:** With the transition from the contemporary (cTnI) to high-sensitivity troponin assay (hs-cTnI), concerns have arisen regarding the diagnostic differences between these two assays due to analytical distinctions. This study aims to evaluate the age and sex differences between these two assays, as well as the differences resulting from using two different 99th percentile values of the high-sensitivity troponin assay. **Method**: A retrospective observational study was conducted at an academic medical center, encompassing a total of 449 lithium heparin plasma samples included in the dataset. Both contemporary and high-sensitivity troponin were simultaneously measured using Siemens ADVIA Centaur analyzers. Two sets of sex-specific 99th percentile URLs from the Siemens study (cutoff-1) and Universal Sample Bank data (cutoff-2) were used for the data analysis. **Results**: The use of cutoff-1 or cutoff-2 had a negligible impact on troponin classification. Troponin elevation significantly increased in individuals > 50 years old for males and >40 years old for females, with both troponin assays. A receiver operating characteristic analysis did not find significant differences between the two assays. The Kaplan–Meier curves showed no differences in survival in cTnI according to the non-sex-specific 99th URL or hs-cTnI (cutoff-2) but showed a slight difference in survival in hs-cTnI (cutoff-1). **Conclusions**: Overall, there were no significant differences in age and sex in the diagnostic performance between the contemporary and high-sensitivity troponin assays. Selection criteria for the establishment of the 99th percentile URL should be standardized to avoid the misinterpretation of the troponin results.

## 1. Introduction

Acute myocardial infarction (AMI) remains a significant contributor to global mortality and morbidity rates. Approximately 10% of all emergency department (ED) consultations are prompted by patients exhibiting symptoms suggestive of AMI [1]. Given the potentially life-threatening nature of AMI, rapid and accurate identification is crucial for accelerating early intervention and effective management strategies.

Cardiac troponins (cTn) I and T are heart-specific proteins, as sensitive and specific biomarkers for detecting myocardial damage [2]. These biomarkers, in conjunction with a 12-lead electrocardiogram (ECG), complement patient history, and physical examinations have been used as common practice in the assessment of patients presenting with acute chest pain [3,4]. Despite therapeutic advancements, the incidence of acute myocardial infarction and associated mortality rates remain significant in patients with acute coronary syndrome (ACS). The differences in ST-segment elevation myocardial infarction (STEMI) and non-ST-segment elevation myocardial infarction (NSTEMI) cases can be partially explained by various factors. For example, older patients and females may present with more subtle or atypical symptoms of NSTEMI, leading to an underdiagnosis [5]. Additionally, the unavailability of highly sensitive cardiac biomarkers may further contribute to the underdiagnosis of NSTEMI cases. Therefore, cardiac troponin assays play a crucial role in establishing the diagnosis of ACS, especially in patients with inconclusive findings on an ECG, particularly in cases of NSTEMI. Consequently, cardiac troponin assays have become indispensable tools in the diagnosis of NSTEMI. Thus, a timely diagnosis, particularly the early identification or exclusion of AMI, is of utmost importance. The early identification of AMI is crucial as it prevents the premature discharge of patients with AMI who may present with normal initial ECG findings. Moreover, it facilitates the prompt initiation of evidence-based therapies, improving patient outcomes [6]. Conversely, the early exclusion of AMI helps avoid unnecessary hospital admissions and enables the expedited discharge of patients, thereby reducing the burden on healthcare resources [4]. Furthermore, delays in either ruling in or ruling out AMI can have disadvantageous consequences. A delayed diagnosis may increase the risk of complications and mortality, particularly in patients with pre-existing coronary artery disease (CAD) [4]. Similarly, delayed assessments and unnecessary investigations resulting from delayed rule-out of AMI can exacerbate patient anxiety, contribute to overcrowding in emergency departments (EDs), and place additional strain on healthcare facilities.

A limitation of contemporary cTn assays is their delayed increase in circulating levels, which results in relative low sensitivity during the initial evaluation of patients with acute chest pain [3,4], which might delay the diagnosis and ultimately delay the management [7]. Over the past decade, high-sensitivity cardiac troponin assays have been developed and integrated into clinical practice. This assay can detect minimal troponin concentrations with high precision, enabling the earlier identification of both high- and low-risk patients for acute myocardial infarction (AMI). Studies have shown that more sensitive cTn assays can enhance the accuracy of an acute myocardial infarction (AMI) diagnosis when patients arrive at the ED [8].

According to the recommendations of The Fourth Universal Definition of Myocardial Infarction (UDMI) and the 2021 American Heart Association (AHA) guidelines for the evaluation and diagnosis of chest pain, high-sensitivity cardiac troponin (hs-Tn) is the preferred biomarker for the detection of myocardial injury, including rule-in and rule-out of AMI [3,4]. The new AHA guidelines and UDMI also recommend the 99th percentile upper reference limits (URLs) as the threshold for defining myocardial injury [3,4]. These recommendations were further solidified by the guidelines on the implementation of hs-cTn assays published in 2018 by the Academy of the American Association for Clinical Chemistry (AACC) and the International Federation of Clinical Chemistry and Laboratory Medicine (IFCC) Task Force on the Clinical Application of Cardiac Biomarkers and other experts [9]. However, there are still concerns that enhancing analytical sensitivity may compromise specificity, despite the widespread adoption of high-sensitivity cardiac troponin (hs-cTn) assays for diagnosing AMI through single or serial measurements of troponin levels ≥ the 99th percentile.

Cardiac troponin results and clinical evaluation are the most crucial for the early assessment of patients with suspected acute coronary syndrome (ACS). Recently, with the development of high-sensitivity cardiac troponin (hs-cTn) assays, the analytical advancements allow the detection of cardiac troponin at very low levels and subsequently the detection of small changes in cardiac troponin [10,11,12,13], compared to the contemporary cardiac troponin assays. There are a few publications, including assay-specific diagnostic algorithms, reporting excellent sensitivities (99.1% to 100%) to rule out AMI and high specificities (94.1–96%) for ruling in AMI using the high-sensitivity troponin assay on Siemens ADVIA Centaur [14,15]. In addition, the transition from contemporary to high-sensitivity troponin assays has not led to an increase in poor outcomes at the emergency department, even though there was the proportional bias observed between these two assays [16]. In a recent study assessing the prognostic value of high-sensitivity cardiac troponin I in patients with suspected AMI using both a conventional cTnI assay and an established hs-TnI assay, the authors reported no differences between the two assays in predicting adverse events. This finding aligns with several other studies that have reported similar results [17].

Studies have demonstrated that hs-cTnI values are significantly lower in females than males [3,18,19,20]. In a large study, the 99th percentile URL of high-sensitivity troponin T increased with age over 60 years, especially in males, with significant differences in the 99th percentile URL of high-sensitivity troponin T based on age and sex specificities [20]. In addition to body composition and left ventricular mass, several other mechanisms have been proposed to explain sex-specific differences, including variations in rates of cardiomyocyte apoptosis due to cardiac remodeling, different myocardial responses to ischemia and reperfusion, or varying degrees of coronary atherosclerosis [21]. Thus, sex and age are recommended to be included in result interpretation while the single threshold value might lead to an overdiagnosis of myocardial infarction. However, despite these proposed mechanisms, several large studies have not shown improved diagnostic accuracy when using sex-specific thresholds [22], and it remains unknown whether the differences between the contemporary and high-sensitivity troponin assays impact the diagnosis and evaluation in patients by age and sex, with sex-specific 99th percentile URLs of high-sensitivity troponin assays.

Another concern is using the 99th percentile URL as a threshold because different 99th percentile URLs have been established based on different study cohorts. According to the FDA approval document for the Siemens ADVIA Centaur high-sensitivity troponin assay, the cutoff of 37 ng/L for females and 57 ng/L for males is provided as 99th percentile URLs, based on data from 2010 on apparently healthy individuals from the United States who ranged between 22 and 91 years of age (50% females and 50% males) [23]. Using the same assay, in 843 healthy volunteers from the United States between 19 and 91 years of age (49% females and 51% males), the 99th percentile of 40 ng/L for males and 26 ng/L for females was reported [24]. Since the sex representation in these two study cohorts was very close, one potential reason for the variation in 99th percentiles URLs might be the different representation of ages or races in the reference populations.

This study aimed to evaluate the age and sex differences when transitioning from contemporary to high-sensitivity troponin on Siemens ADVIA Centaur in hospitalized patients, with two different sex-specific 99th percentile URLs of the same high-sensitivity troponin assays. Additionally, to address concerns regarding the 99th percentile, clinical differences between two different cutoffs for hs-cTnI and the prognostic value of a higher troponin threshold for contemporary cTnI and hs-cTnI assays were also examined.

## 2. Methods

A retrospective observational study was performed at an academic medical center from February to April 2020. This study was approved by the Institutional Review Board of the Ohio State University Wexner Medical Center (OSUWMC, IRB# 2020H0383). Blood samples were collected from an unselected, hospitalized patient cohort. A total of 449 lithium heparin plasma samples were included in the dataset. Demographic information, including age and sex, was obtained from the electronic medical records.

Patients for whom a troponin test was ordered as the standard of care were analyzed using the contemporary ADVIA Centaur TnI-Ultra (cTnI) (Siemens Healthcare Diagnostics, Inc., Tarrytown, NY, USA) and reported in the patient electronic medical record. When available, these samples were simultaneously analyzed using the ADVIA Centaur High-Sensitivity Cardiac Troponin I (hs-cTnI) assay (Siemens Healthcare Diagnostics, Inc., Tarrytown, NY, USA). Troponin results generated using the hs-cTnI assay were not reported to the care team.

The LOQ was 0.03 µg/L (30 ng/L) for the cTnI assay and 2.5 ng/L for the hs-cTnI assay. The 99th percentile URL for cTnI was 0.04 µg/L (40 ng/L). The manufacturer provided 99th percentile URLs for hs-cTnI that were 57 ng/L for males and 37 ng/L for females, referred to as cutoff-1 (Siemens package insert reference). A second set of sex-specific cutoffs for hs-cTnI was also included in the analysis (cutoff-2). Cutoff-2 values were derived from the Universal Sample Bank data and represent the sex-specific 99th percentiles in the United States: 40 ng/L for males and 26 ng/L for females [24]. To further compare across grouped troponin assays, we normalized values to the 99th percentile URL of each individual assay and presented the results as ratios. We further categorized troponin ratios as no elevation (<1 × 99th URL), mild elevation (≥1 to <3 × 99th URL), and severe elevation (≥3 × 99th URL).

A receiver operating characteristic curve (ROC curve) (Graphpad Software, version 8.4.2) was used to estimate the diagnostic accuracy of both contemporary and high-sensitivity troponin assays. A Kaplan–Meier survival analysis (Graphpad Software, version 8.4.2) was run to evaluate the performance of elevated troponin as a prognostic indicator for contemporary cTnI and hs-cTnI assays.

## 3. Results

**Figure 1:**
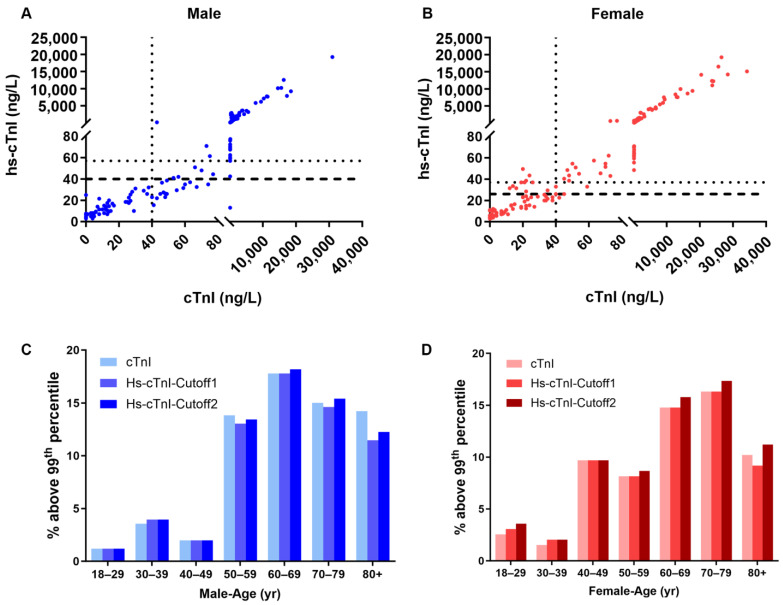
The data distribution of cTnI and hs-cTnI of paired samples. (**A**) Male patients; (**B**) female patients. The dotted horizontal line indicates hs-cTnI cutoff-1; the dashed horizontal line indicates hs-cTnI cutoff-2. The dotted vertical line indicates the 99th percentile URL for cTnI. (**C**) Distribution of troponin results above the 99th percentile URL by ages (male); (**D**) distribution of troponin results above the 99th percentile URL by ages (female). Male population, *n* = 253; female population, *n* = 196.

This study population consisted of 449 samples collected from 253 males (median age: 66 years old) and 196 females (median age: 63 years old) (demographic characteristics shown in Table 1). Figure 1A,B show the distribution of troponin values for the 449 samples analyzed using both the contemporary cTnI assay and the hs-cTnI assay. In the male population, 73% of cTnI results were above the 99th percentile URL, with 66% of Hs-cTnI results exceeding the 99th percentile URL, regardless of whether cutoff-1 or cutoff-2 for Hs-cTnI was utilized. Among females, 63% of cTnI results exceeded the 99th percentile URL, while 68% of HS-cTnI results surpassed this threshold, regardless of the application of cutoff-1 or cutoff-2 for Hs-cTnI (Table 2). The classification of troponin values as either elevated or normal was similar between assays for both males and females (Table 3), with 90% of males and 99% of females with hs-cTnI values > the 99th percentile URL concurrently having cTnI values > the 99th percentile URL. The use of cutoff-1 or cutoff-2 had a negligible impact on troponin classification when evaluating all 449 samples together.

To evaluate the potential impact of sex and age on both troponin assays, the study cohort was divided by sex and age to assess the incidence of increased troponin in both assays. Figure 1C,D show that troponin elevation significantly increased in individuals aged > 50 years old for males and >40 years old for females, in both troponin assays.

**Figure 2:**
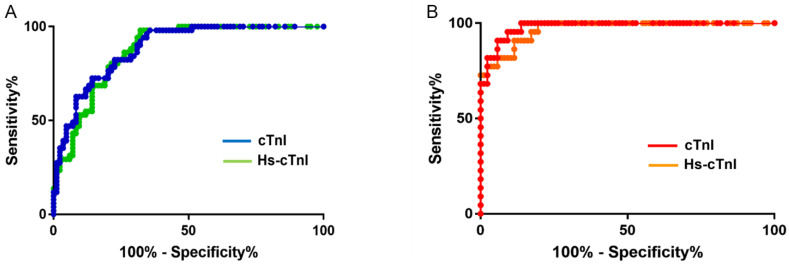
Receiver operating characteristic analysis in patients with NSTEMI. (**A**) Male, *n* = 51 of NSTEMI with both cTnI and hs-cTnI results; (**B**) female, *n* = 22 of NSTEMI with both cTnI and hs-cTnI results.

The receiver operating characteristic analysis was employed to compare the diagnostic ability of contemporary cTnI and hs-cTnI assays in patients with NSTEMI (Figure 2). No significant differences were noted for the area under the curve (AUC) values for males and females using contemporary cTnI or hs-cTnI (males at 0.873 for Hs-cTnI vs. 0.884 for cTnI, and females at 0.969 for Hs-cTnI vs. 0.981 for cTnI).

**Figure 3:**
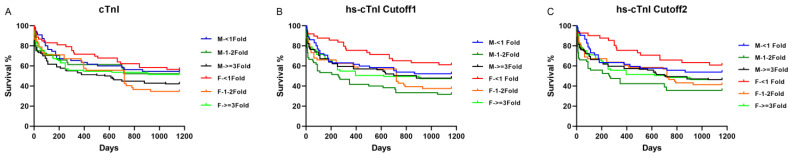
Kaplan–Meier curve analyses stratified by normal, mildly elevated, and severely elevated levels of both cTnI and hs-cTnI assays. (**A**) cTnI assays; (**B**) hs-cTnI cutoff-1 (hs-cTnI1); (**C**) hs-cTnI cutoff-2 (hs-cTnI2).

To evaluate the performance of elevated troponin at different degrees, a fold change of troponin assays was used for the next analysis. We categorized troponin ratios as follows: no elevation (<1 × 99th URL), mild elevation (≥1 to <3 × 99th URL), and severe elevation (≥3 × 99th URL). In both male and female groups for all included patients at 1160 days (Figure 3), the Kaplan–Meier curves showed no differences in survival in cTnI according to the non-sex-specific 99th URL or hs-cTnI according to the sex-specific 99th URL from the Universal Sample Bank data, across the <1-, 1–2-, and ≥3-fold-change groups in both male and female groups.

Interestingly, there were statistical significances in survival in the hs-cTnI group according to the sex-specific 99th URL from the Siemens package insert: male: < 1 fold of 99th URL vs. male: 1–2 fold of 99th URL (Log-rank test *p* = 0.0147, Hazard Ratio (HR) = 0.547, 95% confidence interval (CI) of ratio: 0.3425–0.8734); male: 1–2 fold of 99th URL vs. male: ≥3 fold of 99th URL (Log-rank test *p* = 0.0302, HR = 1.533, 95% CI = 1.007–2.336); and female < 1 fold of 99th URL vs. female 1–2 fold of 99th URL (Log-rank test *p* = 0.0147, HR = 0.5106, 95% CI = 0.2995–0.8707).

## 4. Discussion

The comparison between non-sex-specific contemporary and sex-specific high-sensitivity troponin assays represents a significant contribution to the ongoing discussion about troponin testing in cardiovascular disease diagnoses and prognoses. In this study, we provide a head-to-head comparison of the non-sex-specific contemporary and sex-specific high-sensitivity troponin assays on the Siemens ADVIA Centaur platform, both of which were cleared by the FDA, in hospitalized patients at a single medical center. Our data suggest that there were overall no significant age or sex differences between the contemporary and high-sensitivity troponin assays in the study cohort, despite their analytical differences. When using two different sex-specific 99th percentile URLs for the high-sensitivity troponin assay based on different reference populations, we found significant differences in the prognosis in males when categorizing hs-cTnI as fold changes of the 99th percentile URL. Similar differences were observed in females.

Sex is one of the various factors that could affect troponin concentration and interpretation, potentially resulting in an underdiagnosis and disparities in the treatment of AMI in women. Initially, the first cTn assays required the utilization of a singular, standardized cutoff value [25]. However, the advent of high-sensitivity troponin assays, with improving analytical sensitivity, has revealed that men exhibit notably higher concentrations than women for both hs-cTnT and hs-cTnI. This underlines the possibility that the upper reference limit for diagnosing MI may be twice as high in men compared to women, regardless of the assay used [13,20,25]. The debate surrounding the utilization of sex-specific 99th percentile upper reference limits (URLs) for high-sensitivity troponin assays persists within the scientific community. Prior research has demonstrated lower values of high-sensitivity cardiac troponin in females compared to males [3,18,19,20], prompting discussions about the need for sex-specific thresholds to mitigate the risk of an underdiagnosis, especially in female patients. Several hypotheses have been proposed to explain these differences, including variations in cardiac physiology and responses to physiological stressors. One explanation is that this difference might be due to the larger left ventricular mass in males, leading to the proposal of sex-specific 99th percentile URLs for hs-cTn assays to prevent an underdiagnosis of myocardial infarction in females [3,20]. Other potential explanations include different rates of cardiac remodeling resulting in varying degrees of cardiomyocyte apoptosis [26,27], different responses of cardiac myocytes to physical activity [28], and protective effects against oxidative damage by estrogens [29,30]. However, the use of sex-specific 99th percentile URLs remains controversial, as some large studies failed to prove improved diagnostic accuracy in myocardial infarction [22] or additional prognostic performance for risk predictions [31] with sex-specific 99th percentile URLs for troponin. In a multicenter study, the diagnostic efficacy of a high-sensitivity troponin assay was compared with that of a contemporary troponin assay in suspected acute coronary syndrome (ACS) cases. Initially, clinical decisions were based on cTnI values alone, with hs-cTnI concentrations obscured. Subsequently, during the second phase, clinicians were provided with hs-cTnI levels while cTnI values remained undisclosed. Sex-specific 99th percentile cutoffs were applied for the hs-cTnI assay. There were no significant differences in 1-year outcomes observed among patients reclassified based on cTnI versus hs-cTnI levels [32]. Another study found that utilizing sex-specific 99th percentile cutoffs for hs-cTnI enhances the identification of women at high risk for cardiovascular events within 1 year. However, the overall impact across the entire ED population with chest pain symptoms would likely be insignificant [33]. In our study, we observed that the age of elevated troponin was about 10 years earlier in females than in males, but we did not find significant differences in sex between the contemporary and high-sensitivity troponin assays in the diagnosis of NSTEMI, which is consistent with the previous finding.

We noticed a slight difference in the prognostic evaluation with the two different 99th percentile URLs for the same high-sensitivity troponin assay in our study, highlighting the importance of standardized methodologies in establishing reference thresholds. This result is not surprising because even an entirely normal ECG would not likely reveal pre-existing myocardial damage [34] when screening healthy individuals, despite variations in questionnaires and choices of other biomarkers. Thus, the selection criteria for healthy individuals greatly affect the determination of the 99th percentile value, resulting in a lower 99th percentile URL when using rigorous selection criteria and a higher 99th percentile URL when using less strict criteria. Since the 99th percentile thresholds can vary significantly depending on the cohort selection [24,25,35], the 2022 International Federation of Clinical Chemistry and Laboratory Medicine (IFCC) and American Association of Clinical Chemistry guidelines recommend that the 99th percentile thresholds should be derived from a sample size of at least 400 males and 400 females, with biomarkers used to exclude people with subclinical disease [36]. The recent guidelines from the International Federation of Clinical Chemistry and Laboratory Medicine and the American Association of Clinical Chemistry [9] emphasize the importance of consistent approaches in determining these thresholds to ensure reliability and comparability across studies.

While our study provides valuable insights into troponin assay performance, it is essential to acknowledge its limitations. Our patient cohort included a small number of hospitalized patients with troponin testing ordered for potential suspected cardiovascular diseases. It does not reflect the entire spectrum of patients presenting with a suspicion of acute coronary syndrome in the United States. However, given the minimal differences between the contemporary and high-sensitivity troponin assays on Siemens Centaur, further replication with the contemporary troponin assay is warranted for the commercially available high-sensitivity troponin assay. Replication studies using larger and more diverse patient populations would provide valuable insights into the comparative performance of contemporary and high-sensitivity troponin assays in real-world clinical settings.

In conclusion, our study underlines the ongoing debate about the use of sex-specific 99th percentile URLs for high-sensitivity troponin assays and highlights the importance of standardized methodologies in their determination. Our finding in a single center does not support the effect of the sex-specific 99th percentile cutoff value of hs-cTnI in the diagnosis and prognosis performance. Therefore, caution of implementing the sex-specific 99th percentile cutoff value is essential due to the insufficient data on pathophysiology, and additional studies are necessary to elucidate whether and how the implementation of sex-specific cutoffs could improve the management of ACS or AMI in females.

## Figures and Tables

**Table 1:** Demographic characteristics of study population.

Age [Median (Years Old)]	NSTEMI	
**Male**	66 (24–92) (*n* = 253)	51 (20.2%)
**Female**	63 (25–98) (*n*=196)	22 (11.2%)

**Table 2:** Data distribution of hs-cTnI results.

	Contemporary cTnI	High-Sensitivity cTnI (Cutoff-1)	High-Sensitivity cTnI (Cutoff-2)
**LoQ**	30 ng/L	2.5 ng/L	
**99% URL (** **Male)**	40 ng/L	57 ng/L	40 ng/L
**Above 99% URL (Male)**	185/253 (73%)	168/253 (66%)	168/253 (66%)
**99%** **URL (Female)**	40 ng/L	37 ng/L	26 ng/L
**Above 99% URL (Female)**	123/196 (63%)	134/196 (68%)	133/196 (68%)

**Table 3:** Concordance of Hs-cTnI results and cTnI results.

	**Hs-cTnI above 99% URL (Cutoff-1)**	**Hs-cTnI above 99% URL (Cutoff-2)**
**cTnI above 99% URL (Male) (*n* = 185)**	167/185 (90%)	167/185 (90%)
**cTnI above 99% URL (Female) (*n* = 123)**	122/123 (99%)	122/123 (99%)
	**Hs-cTnI below 99% URL (Cutoff-1)**	**Hs-cTnI below 99% URL (Cutoff-2)**
**cTnI below 99% URL (Male) (*n* = 68)**	67/68 (99%)	67/68 (99%)
**cTnI below 99% URL (Female) (*n* = 73)**	61/73 (84%)	62/73 (85%)

## Data Availability

The original contributions presented in the study are included in the article.
